# Classical and γδ T cells are each independently sufficient to establish protection against a classical strain of *Klebsiella pneumoniae*

**DOI:** 10.3389/fcimb.2022.974175

**Published:** 2022-08-31

**Authors:** Joseph J. Mackel, Catherine Morffy Smith, Rachel K. Wasbotten, Joy Twentyman, David A. Rosen

**Affiliations:** ^1^Department of Pediatrics, Division of Infectious Diseases, Washington University School of Medicine, Saint Louis, MO, United States; ^2^Department of Molecular Microbiology, Washington University School of Medicine, Saint Louis, MO, United States

**Keywords:** classical *Klebsiella pneumoniae*, pulmonary immunity, T cells, convalescent serum transfer, adaptive immunity

## Abstract

Infections with classical strains of the Gram-negative bacterium *Klebsiella pneumoniae* pose a significant clinical challenge due to rising antibiotic resistance. We previously established a lung inoculation plus challenge model using live, classical *K. pneumoniae* in order to study host protection. Here, we employ this model to dissect adaptive immune responses to this critical pathogen. First, we performed convalescent serum transfers from inoculated mice to naïve recipients and found that classical *K. pneumoniae* infection outcomes, unlike hypervirulent *K. pneumoniae* infection outcomes, were not improved. This suggests that circulating antibody responses alone are not sufficient to mediate protection against this classical strain. Hence, we evaluated the role of T cells in protection against classical *K. pneumoniae* reinfection and demonstrated that mice lacking T cells are unable to establish a protective response. However, mice individually deficient in either of the major T cell subsets, γδ or αβ (classical T cells), effectively mount a protective response, indicating either subset alone is sufficient to mediate protection. Sequestration of T cells in secondary lymphoid organs during the challenge infection did not ablate protection, indicating the circulating T cell pool is not required for the protective phenotype. Finally, we demonstrate that depletion of T cells during initial infection eliminates protection against challenge. Collectively, these experiments demonstrate the imperative contribution of T cells to protective immunity against classical *K. pneumoniae* and will guide further inquiries into host effector responses required to control this infection.

## Introduction

As the rates of antibiotic-resistant infections rise, strategies to prevent and treat these infections are a critical public health priority. The Gram-negative bacterial species *Klebsiella pneumoniae* (Kp) is the third leading cause of antimicrobial-resistance (AMR) disease burden, with over 250,000 deaths attributable to AMR Kp worldwide in 2019 (Murray et al., 2022). The Centers for Disease Control has identified Kp as an urgent threat and research priority among drug-resistant organisms (CDC, 2019). Two pathotypes of Kp, classical (cKp) and hypervirulent (hvKp), cause infections in humans and exhibit substantially different virulence characteristics and pathogenesis. Classical strains commonly cause pneumonia, urinary tract infection, and bacteremia in medically complicated or immunocompromised patients, whereas hypervirulent strains often infect immunocompetent individuals, causing pyogenic liver abscess and other disseminated infections (Paczosa and Mecsas, 2016; Gonzalez-Ferrer et al., 2021). Development of novel immune-based strategies targeting specific pathogens such as Kp is a promising approach to the challenge of AMR but requires detailed understanding of host immune responses specific to these infections.

Antibody responses raised against hvKp are sufficient for protection against autologous hvKp challenge (Chen et al., 2011). In the absence of antibody, Th17 cells elicited by heat-killed hvKp inoculation of the lung promote bacterial clearance during subsequent pulmonary challenge with the initial inoculation strain or a heterologous classical strain (Chen et al., 2011). Further, heat-killed hvKp inoculation establishes a population of lung resident T cells derived from Th17 cells that promote clearance of Kp from the lung during challenge (Amezcua Vesely et al., 2019). Conversely, little is understood about the immune responses elicited by infection with live cKp and how these responses protect from reinfection. Our laboratory recently developed a model of inoculation plus challenge using live cKp (Twentyman et al., 2020). In the current work, we dissect the cell populations established in survivors of lung infection with live cKp and the contributions of these responses to protection from subsequent challenge. We demonstrate that T cells are required for adaptive immunity to cKp and that circulating antibody alone does not protect against cKp pneumonia. Furthermore, either γδ or classical αβ T cells are sufficient for protection. Together, these studies contribute to the foundational understanding of the host response to live cKp lung infection that may inform development of targeted immunotherapeutics against cKp infection.

## Material and methods

### Bacterial preparations

Infections were performed with the cKp strain TOP52 (Rosen et al., 2008; Johnson et al., 2014; Wylie et al., 2019; Twentyman et al., 2020) or hvKp 43816 (Bakker-Woudenberg et al., 1985). Bacteria were grown statically in 20-mL cultures of Luria-Bertani (LB) broth overnight at 37°C. Cultures were centrifuged at 8000 × *g* for 10 min, and bacteria were subsequently resuspended in sterile PBS and diluted to the desired inoculum concentration by measuring optical density at 600 nm (OD_600_). Inocula were verified by serial dilution and plating. For inoculations utilizing 43816, bacteria were heat-killed at 60°C for 30 min, and lack of viability was confirmed *via* plating.

### Mouse infections

All animal procedures complied with ethical regulations for animal testing and research and were approved by the Institutional Animal Care and Use Committee at Washington University School of Medicine. Mice aged 7-8 weeks at the initiation of each experiment were used for all studies. All mouse strains were initially acquired from The Jackson Laboratory: C57BL/6J (000664), *Tcrb*^-/-^*Tcrd*^-/-^ (B6.129P2-Tcrb^tm1Mom^ Tcrd^tm1Mom^/J; 002122), *Tcrb*^-/-^ (B6.129SP2-*Tcrb^tm1Mom^*/J; 002118), and *Tcrd*^-/-^ (B6.129P2-Tcrd^tm1Mom^/J; 002120), and mutant strains were further bred in Washington University animal facilities. Except where otherwise noted, mice were infected *via* surgical intratracheal instillation with 1-2 x 10^7^ CFU, as previously described (Twentyman et al., 2020). Briefly, mice were anesthetized with inhaled isoflurane and the trachea exposed by surgical dissection. Inoculum (20 μL volume) was injected intratracheally using a 30-gauge, caudally-directed needle. Overlying tissues were replaced and skin was closed using Vetbond (3M Animal Care Products). Mice received 1 mg/kg of buprenorphine SR subcutaneously for pain control. Certain experiments, as indicated, used a non-surgical oropharyngeal aspiration method (Wasbotten et al., 2022) in which mice were anesthetized with inhaled isoflurane and suspended by the upper incisors. The tongue was then retracted laterally and inoculum (50 μL volume) was administered *via* pipet to the oropharynx and aspirated on subsequent breath.

For survival experiments, mice were monitored for morbidity/mortality and weighed daily. Mice falling under 75% of their initial weight for two consecutive days were humanely euthanized. After 28 days, surviving mice (now aged 11-12 weeks) were challenged using the same method as in the primary infection. Mortality and weight changes were then assessed for an additional 14 days prior to sacrifice.

### Flow cytometry

For flow cytometric analyses, lungs were perfused and removed, placed in sterile PBS, then transferred to 1 mL digestion media [2.5 mg/mL Collagenase D (Roche 11088866001) + 3% fetal bovine serum (FBS; Gibco 10438-026) in RPMI medium (Sigma R8758)], minced with scissors, and incubated for 1 h at 37°C with shaking at 200 rpm. Digested lung pieces were dissociated through 40-μm cell strainers and washed with flow cytometry buffer [PBS + 0.5% BSA (Sigma A7030) + 2 mM EDTA]. All centrifugation steps were performed in a swinging bucket centrifuge at 300 × *g*. Red blood cells were lysed with Pharm Lyse Buffer (BD Biosciences 555899). Cells were washed, then resuspended in FACS buffer [PBS + 1% FBS (Gibco 10438-026) + 0.9% sodium azide], blocked with Fc Block (BD Biosciences 553142) for 10 min, and stained at 4°C for 30 min with CD45-BV510 (103138), CD3-APC/Cyanine7 (100222), CD4-FITC (116004), TCRγ/δ-PE (118108), CD69-BV421 (104545), from Biolegend, and CD11b (557960), Ly6G-FITC (551460), and CD8-PeCy7 (552877) from BD Biosciences. After staining, cells were washed then fixed in 2% paraformaldehyde until acquisition on a Benton Dickinson (BD) LSR II Fortessa cytometer. Total cell counts per organ were calculated using Precision Count Beads (Biolegend 424902) according to the manufacturer’s instructions.

### Convalescent serum transfer

Twenty-eight days following inoculation with either live cKp TOP52 or heat-killed hvKp 43816, mice were anesthetized with 3% isoflurane and terminally bled *via* cardiac puncture. Sera were isolated with Microtainer SST tubes (BD 365967), pooled, and 500 μL was injected intraperitoneally (i.p.) into each naïve recipient. Mice were then rested overnight before challenge with live bacteria matched to the convalescent serum administered. In experiments to quantify bacterial burdens, sera were obtained from donor mice inoculated with 1-2 x 10^8^ CFU live cKp, 1-2 x 10^8^ CFU heat-killed hvKp, or PBS *via* oropharyngeal aspiration; recipient mice were challenged with 1-2 x 10^8^ CFU live cKp or 1-2 x 10^6^ CFU live hvKp *via* oropharyngeal aspiration. Lungs and spleens were collected into PBS at 24h post infection, homogenized, and plated to determine organ bacterial titers. For survival studies, sera were obtained from donor mice inoculated with 1-2 x 10^7^ CFU live cKp, 1-2 x 10^7^ CFU heat-killed hvKp, or PBS *via* intratracheal instillation; recipient mice were challenged with 1-2 x 10^7^ CFU live cKp or 2 x 10^3^ CFU live hvKp *via* intratracheal instillation.

### FTY720 treatments

The sphingosine-1- phosphate receptor agonist FTY720 (Sigma SML0700) was reconstituted to 10 mg/mL in water and stored at -20°C. Mice were inoculated with 1-2 x 10^7^ CFU live cKp or PBS *via* oropharyngeal aspiration. Beginning one day prior to challenge, mice were injected with 20 μg FTY720 i.p. daily throughout the entire 14-day study period. Control mice were injected with 100 μL water. Twenty-eight days following inoculation, mice were challenged with 1-2 x 10^8^ CFU cKp *via* oropharyngeal aspiration.

### *In vivo* T cell depletions

Mice were injected i.p. with anti-CD3 monoclonal antibody (clone 145-2C11) or isotype control Armenian Hamster IgG (Bio X Cell). For T cell depletion at inoculation, mice received 200 μg antibody on day -6 relative to inoculation, followed by 100μg antibody every 4 days through day 14 post-inoculation. For T cell depletion at challenge, mice received 200 μg antibody on day -6 relative to challenge, followed by 100 μg antibody every 4 days through day 10 post-challenge. Additional mice were analyzed for T cell depletion *via* flow cytometry. Mice were inoculated with 1-2 x 10^7^ CFU live cKp or PBS *via* oropharyngeal aspiration. Mice were challenged with 1-2 x 10^8^ CFU live cKp *via* oropharyngeal aspiration.

### Statistics

For Kaplan–Meier survival analyses, the Mantel–Cox log-rank test was used to determine differences in survival between two groups. Student’s *t*-test with Holm-Sidak correction for multiple comparisons was used to determine differences in mouse weights. The Mann–Whitney U-test was used for comparisons between cell counts obtained *via* flow cytometry and organ titers, as they were not all normally distributed. All tests were two-tailed and P-values <0.05 were considered significant. Analyses were performed using GraphPad Prism 9.

## Results

### Convalescent serum transfer is not protective against cKp TOP52 pneumonia

**Figure 1:**
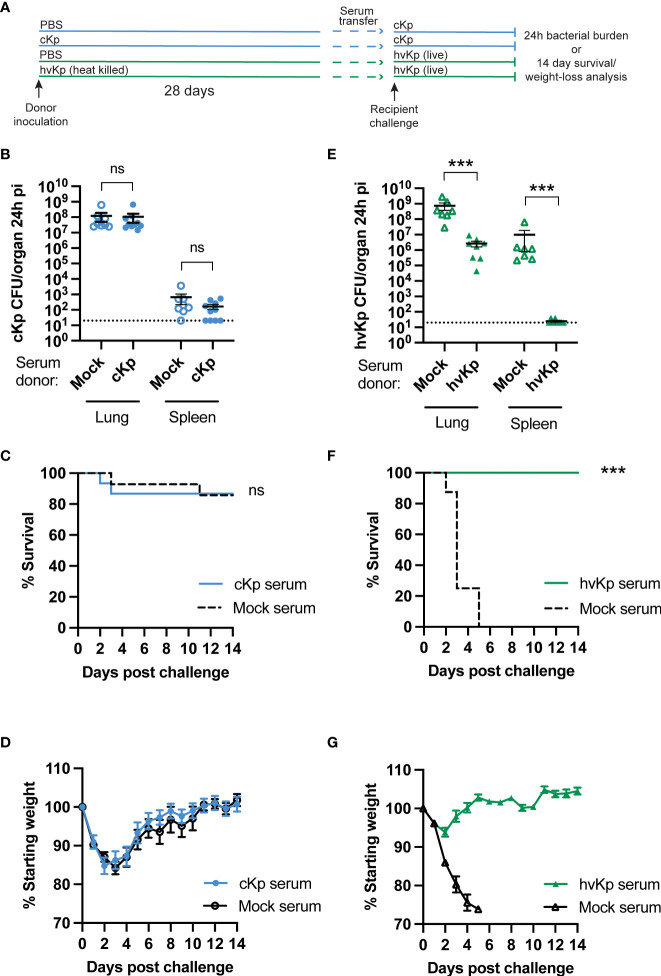
Convalescent serum transfer is not protective against cKp TOP52 infection. **(A)** Scheme of convalescent serum transfer and homologous challenge. **(B)** Lung and spleen burden of anti-cKp serum recipients 24h following cKp challenge with 10^8^ CFU (n=8-10 mice per group, 2 independent experiments). **(C)** Survival and **(D)** weights of anti-cKp serum recipient mice over 14 days following cKp challenge with 10^7^ CFU (n=14-15 mice per group, 2 independent experiments). Mouse weights were not significantly different at any time point. **(E)** Lung and spleen burden of anti-hvKp serum recipients 24h following hvKp challenge with 10^6^ CFU (n=7-8 mice per group, 2 independent experiments). **(F)** Survival and **(G)** weight loss of anti-hvKp serum recipient mice over 14 days following hvKp challenge with 10^3^ CFU (n=8-10 mice per group, 1 experiment). Weights of mice in the mock serum groups were significantly lower than weights of mice receiving anti-hvKp serum on days 2-5 post infection (p< 0.001). For **(F, G)** all mice in the mock serum group died by day 5 post challenge. Bars indicate mean +/- SEM. *** indicates p<0.001. ns indicates not significant. Dotted line indicates assay limit of detection.

Specific antibody responses are protective against many infections including hvKp pneumonia (Diago-Navarro et al., 2017; Feldman et al., 2019). We have previously demonstrated that cKp inoculation elicits a specific serum IgG response (Twentyman et al., 2020), but whether circulating IgG contributes to protection against subsequent challenge is unknown. We transferred convalescent sera from cKp-inoculated donors or PBS-inoculated controls (28 days post inoculation) to naïve recipients (**Figure 1A**). Upon cKp challenge of these recipients, serum transfer from cKp donors did not impact lung or spleen bacterial burden at 24h post infection (**Figure 1B**), survival (**Figure 1C**), or weight loss (**Figure 1D**). In contrast, homologous serum transfer was highly effective at reducing lung burden and preventing dissemination to the spleen in hvKp infection (**Figure 1E**). These results correlated with survival studies, in which mice receiving anti-hvKp serum were protected from mortality (**Figure 1F**) and weight loss (**Figure 1G**) during subsequent homologous hvKp infection. Together, these results indicate that, despite their efficacy against hvKp infection, circulating antibodies are not sufficient to protect against cKp TOP52 lung infection.

### T cells are required for adaptive immunity against cKp pneumonia

**Figure 2:**
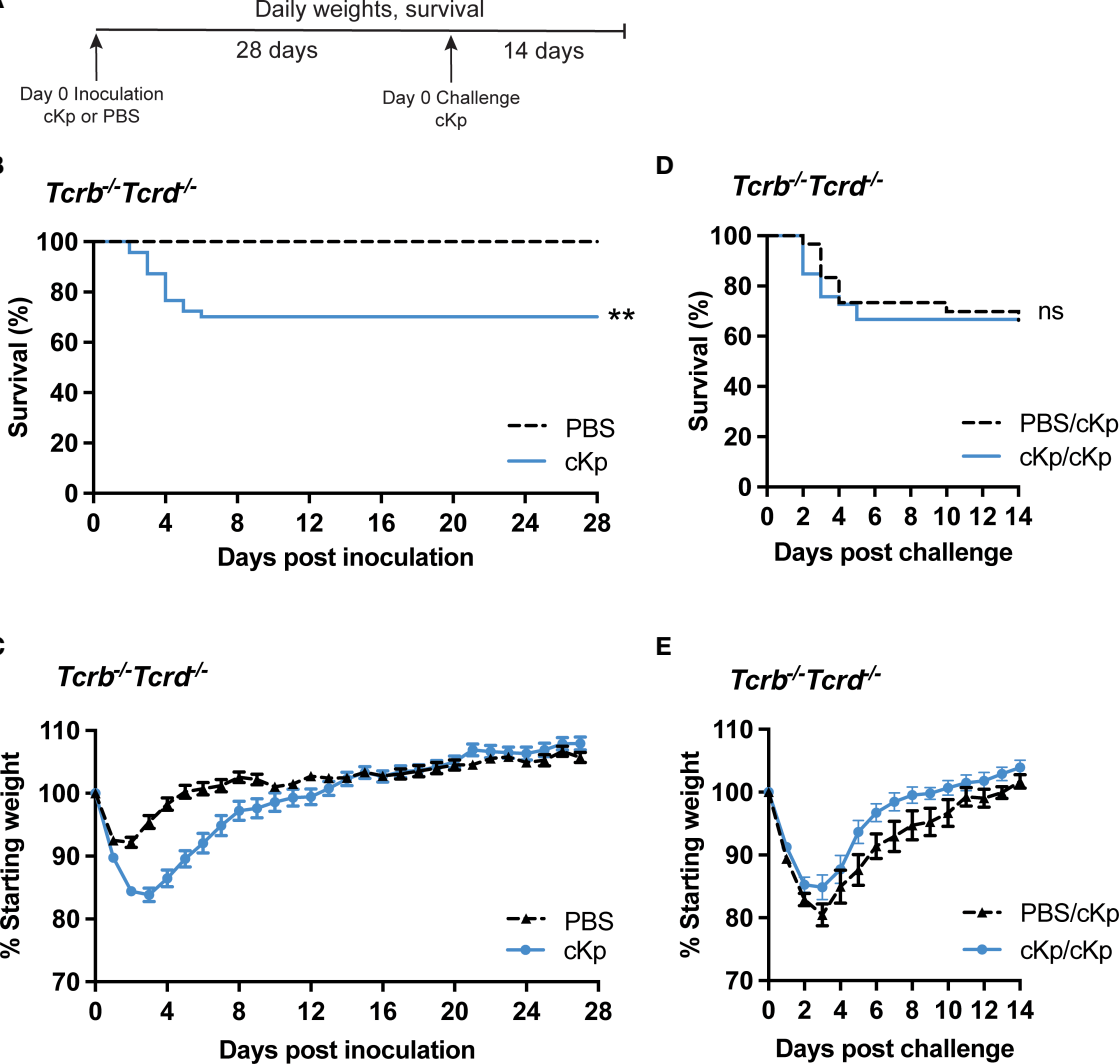
Inoculated *Tcrb*^-/-^*Tcrd*^-/-^ mice are not protected against cKp challenge. **(A)** Scheme of cKp inoculation and challenge in *Tcrb*^-/-^*Tcrd*^-/-^ mice. **(B)** Survival and **(C)** weights of *Tcrb*^-/-^*Tcrd*^-/-^ mice over 28 days following inoculation with 10^7^ CFU cKp (n=48) or PBS (n=30). Weights of mice inoculated with cKp were significantly lower than mock-infected mice on days 2-8 post infection (p<0.05). **(D)** Survival and **(E)** weights of *Tcrb*^-/-^*Tcrd*^-/-^ mice over 14 days following challenge with 10^7^ CFU cKp (PBS/cKp n=30; cKp/cKp n=33). Mouse weights were not significantly different at any time point between the PBS/cKp and the cKp/cKp groups. Bars indicate mean +/- SEM. ** indicates p<0.01. ns indicates not significant. Data are combined from at least 3 independent experiments.

We have previously demonstrated that a RAG1-dependent adaptive immune response (requiring mature B and/or T cells) is established following cKp infection and protects mice from morbidity and mortality during reinfection with the same strain (Twentyman et al., 2020). As the results in **Figure 1** demonstrated that an antibody response was not sufficient to protect from cKp challenge, we hypothesized that T cells are required for such protection. We inoculated mice lacking all T cells (*Tcrb*^-/-^*Tcrd*^-/-^) with cKp TOP52 or PBS (controls), then challenged survivors with cKp after 28 days (**Figure 2A**). Approximately 25% of *Tcrb*^-/-^*Tcrd*^-/-^ mice succumbed to primary infection or were euthanized due to excessive weight loss (**Figure 2B**), while infected mice on average lost 15% of their body weight at the peak of infection (**Figure 2C**). *Tcrb*^-/-^*Tcrd*^-/-^ mice surviving initial cKp infection did not demonstrate protective immunity upon challenge. *Tcrb*^-/-^*Tcrd*^-/-^ survivors exhibited similar survival (**Figure 2D**) and weight loss (**Figure 2E**) during challenge infection as seen in age-matched controls during primary infection. Therefore, unlike wild-type mice in our previous report that were protected from challenge infection (Twentyman et al., 2020), *Tcrb*^-/-^*Tcrd*^-/-^ mice were not protected. These data demonstrate a requirement for T cells in adaptive immunity to cKp lung infection.

### Multiple T cell populations are increased in the lung following cKp infection

**Figure 3:**
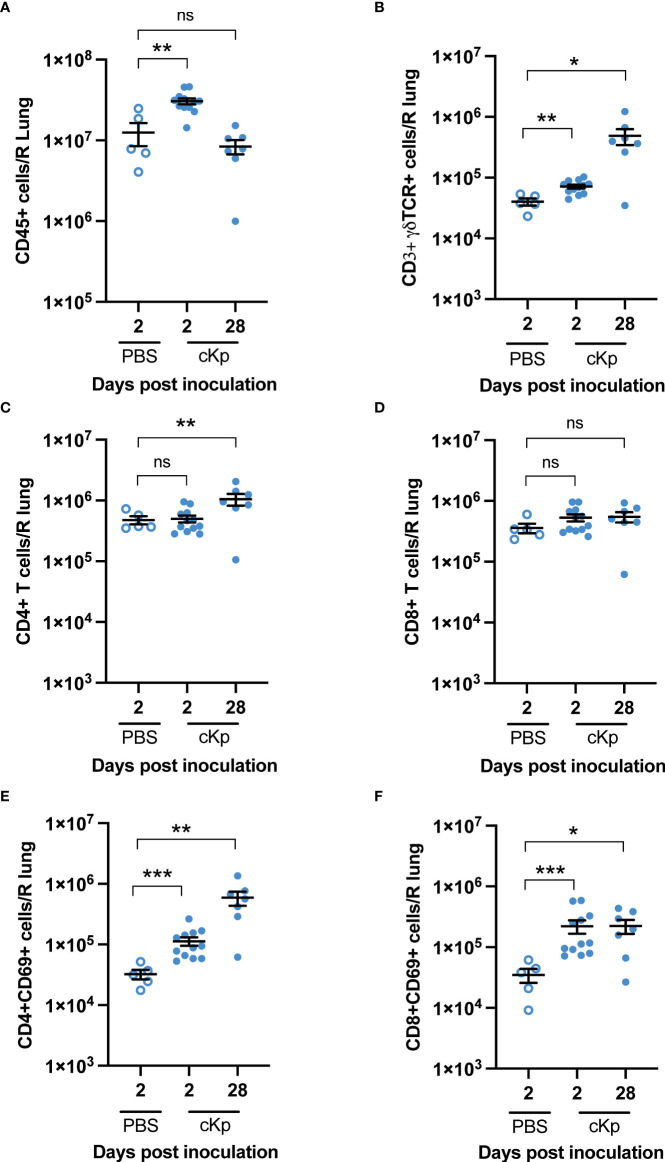
Multiple T cell populations are present in the lung following cKp inoculation. Flow cytometric enumeration of **(A)** CD45+ **(B)** CD3+γδTCR+ **(C)** CD3+CD4+ **(D)** CD3+CD8+ **(E)** CD3+CD4+CD69+, and **(F)** CD3+CD8+CD69+ populations 2 days and 28 days following cKp inoculation *via* oropharyngeal aspiration (10^8^ CFU) or 2 days following PBS inoculation (n=5-12 mice per group, 2 independent experiments). Bars indicate mean +/- SEM. *** indicates p<0.001, ** indicates p<0.01, * indicates p<0.05. ns indicates not significant.

We next assessed if T cell populations are increased within the lung following cKp infection. Flow cytometric analysis indicated an increase in total CD45+ cells in the lung at 2 days post cKp inoculation compared with control mice. This increase in total CD45+ cells primarily reflected neutrophil infiltration (**Figure S1**) and resolved by 28 days post inoculation (**Figure 3A**). We observed a significant increase in the number of γδ T cells in the infected lung at day 28 compared to control mice (**Figure 3B**). The number of CD4 T cells (**Figure 3C**) was increased at 28 days post infection, while the number of CD8 T cells was similar (**Figure 3D**). The numbers of CD4 (**Figure 3E**) and CD8 (**Figure 3F**) T cells expressing CD69, a marker associated with activation and tissue residency, were both increased at day 2 and day 28 post inoculation. The increases in these T cell populations, which persist until at least the time of challenge, suggest they may contribute to the T cell-dependent protection observed in this model.

### Recruitment of T cells during challenge is not required for protection

**Figure 4:**
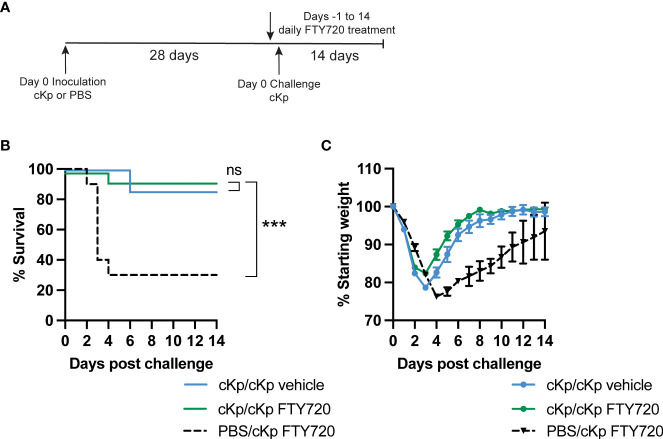
Inhibition of T cell recruitment during cKp challenge does not inhibit protection in cKp-inoculated mice. **(A)** Scheme of cKp inoculation (10^7^ CFU) plus challenge (10^8^ CFU) *via* oropharyngeal aspiration and FTY720 treatment in wild-type mice. **(B)** Survival and **(C)** weights of FTY720 treated mice over 14 days following challenge with cKp (n=10-15 mice per group, 2 independent experiments). Weights of cKp/cKp vehicle-treated mice were significantly lower than weights of cKp/cKp FTY720-treated mice on day 3 post infection (p<0.001). Weights of PBS/cKp FTY720-treated mice were significantly higher than weights of cKp/cKp FTY720-treated mice on days 1-2 and significantly lower on days 4-13 post infection (p<0.01). Bars indicate mean +/- SEM. *** indicates p<0.001. ns indicates not significant.

Previous studies have demonstrated that CD4 T cells mediating clearance of hvKp following inoculation are lung-resident (Amezcua Vesely et al., 2019). Thus, we determined if recruitment of T cells to the lung is required during cKp challenge of previously cKp-infected mice. FTY720 treatment inhibits sphingosine 1-phosphate signaling, thereby sequestering circulating T cells in secondary lymphoid organs (Ledgerwood et al., 2008) and preventing their trafficking to tissues. Wild-type mice that survived survived initial cKp infection and controls were treated with FTY720 beginning one day prior to infection (**Figure 4A**). Analysis of peripheral blood one day following the first treatment (i.e. day of infection) demonstrated greatly decreased T cell numbers in the blood, confirming efficacy of FTY720 treatment at inhibiting T cell circulation (**Figure S2**). FTY720 treatment did not inhibit protection during challenge as measured by survival (**Figure 4B**) and weight loss (**Figure 4C**). These data suggest that the protective immune response to cKp does not require recruitment of T cells to the lung at the time of challenge.

### Either classical or γδ T cells are sufficient for protection

**Figure 5:**
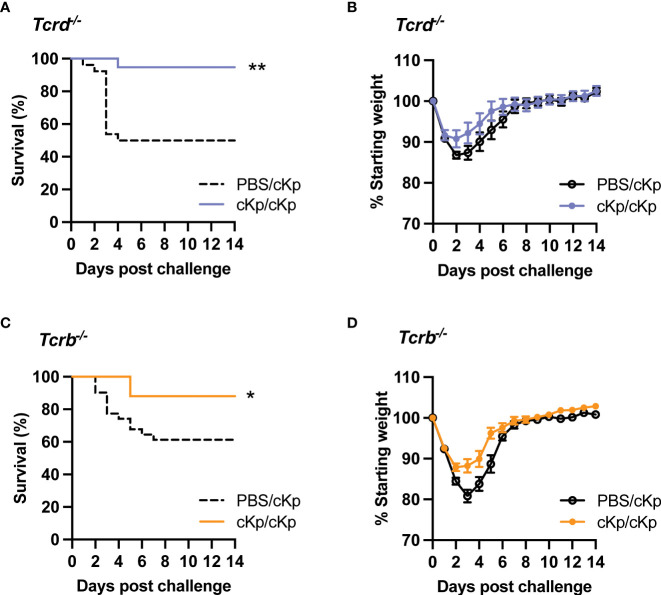
Inoculated *Tcrd*^-/-^ and *Tcrb*^-/-^ mice are each protected against cKp challenge. **(A)** Survival and **(B)** weights of *Tcrd*^-/-^ mice previously-inoculated with PBS or 10^7^ CFU cKp over 14 days following challenge with 10^7^ CFU cKp (PBS/cKp n=26, cKp/cKp n=20). Weights of PBS/cKp *Tcrd^-/-^* mice were not significantly different than weights of cKp/cKp *Tcrd^-/-^* mice at any time point. **(C)** Survival and **(D)** weights of *Tcrb*^-/-^ mice previously-inoculated with PBS or 10^7^ CFU over 14 days following challenge with 10^7^ CFU cKp (PBS/cKp n=31, cKp/cKp n=25). Weights of PBS/cKp *Tcrb^-/-^* mice were significantly lower than weights of cKp/cKp *Tcrb^-/-^* mice on day 3 post challenge (p<0.01). Bars indicate mean +/- SEM. ** indicates p<0.01, * indicates p<0.05. Data are combined from at least 3 independent experiments.

As large populations of γδ T cells as well as CD69+ CD4 and CD8 T cells are established in the lung following inoculation, we sought to identify a potential requirement for these subsets by employing mice deficient in either γδ T cells or classical αβ T cells (including CD4 and CD8 T cells). Mice lacking γδ T cells (*Tcrd*^-/-^) were able to mount a statistically significant protective immune response (**Figures 5A, B**), indicating that γδ T cells are dispensable in the adaptive immune response to cKp. Similarly, mice lacking classical αβ T cells (*Tcrb*^-/-^) were protected against cKp challenge (**Figures 5C, D**), indicating that classical T cells are not required in the anti-cKp adaptive immune response. To further corroborate these findings, we performed additional experiments in which mice were inoculated with 10^7^ cKp and subsequently challenged with a higher dose of 10^8^ cKp and also observed protection from mortality (**Figure S3**). Together with the absence of protection in *Tcrb*^-/-^*Tcrd*^-/-^ mice (**Figure 2**), these experiments in mice individually deficient in γδ T cells or classical αβ T cells indicate that each of these T cell subsets is individually sufficient to mediate a protective adaptive immune response to cKp.

### T cells are required to establish protection against challenge

**Figure 6:**
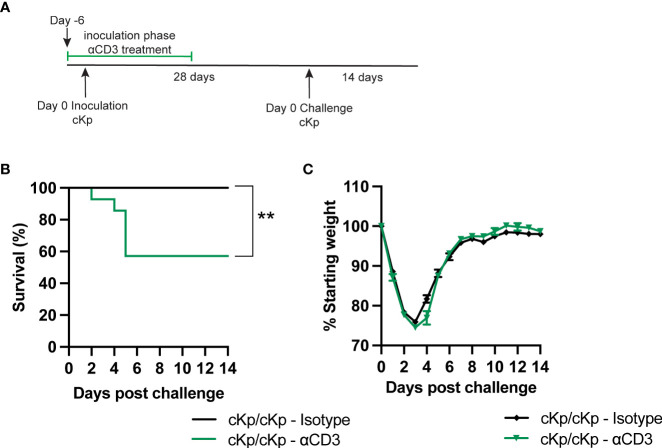
Depletion of T cells at the time of inoculation inhibits protection against cKp challenge. **(A)** Scheme of cKp inoculation (10^7^ CFU) plus challenge (10^8^ CFU) *via* oropharyngeal aspiration and antibody-mediated T cell depletion in wild-type mice. **(B)** Survival and **(C)** weights of anti-CD3 or isotype-treated mice over 14 days following challenge with cKp (n=9-14 mice per group, 2 independent experiments). Weights of anti-CD3-treated mice were not significantly different than weights of isotype-treated mice at any time point. Bars indicate mean +/- SEM. ** indicates p<0.01.

We next sought to determine if depletion of T cells during the inoculation or challenge infections in wild-type animals affected protection. Mice were depleted of T cells *via* treatment with anti-CD3 antibody preceding and during inoculation (**Figure 6A**). Control mice were treated with isotype antibody. Antibody treatment reduced T cell numbers by approximately 80% in the lung and 90% in the spleen in naïve animals prior to inoculation (**Figure S4**). Mice that had been depleted of T cells during primary infection exhibited increased mortality upon challenge, compared to isotype-treated mice (**Figures 6B, C**). We performed additional experiments to deplete T cells only at the time of secondary infection (**Figure S5**). Mice treated with anti-CD3 depleting antibody retained the protective phenotype; however, based on the efficacy of depletion in the lung (**Figure S4**) we cannot entirely exclude a contribution of lung T cells in protection during the time of challenge. Together, these data demonstrate a clear requirement for T cells at the time of inoculation in order to establish a protective immune response.

## Discussion

In the current work, we identify a requirement for T cells in the establishment of protective adaptive immunity against cKp in the lung, which can be met by either αβ or γδ T cells. To our knowledge, this is the first study to define a role for T cells elicited by cKp infection in protection against challenge. Convalescent serum transfer experiments suggest that a circulating antibody response confers inadequate protection against reinfection with cKp; thus, the required function of T cells is not in providing help to B cells to produce an effective IgG response. Multiple T cell populations including γδ T cells and classical T cell populations are established in the lung following cKp inoculation and are independently sufficient to mediate establish protection. These studies characterize the normal host immune response to live cKp lung infection and precipitate further questions regarding the effector functions responsible for protection against cKp in the lung. These data may also extrapolate to pre-existing immune responses in humans who have previously encountered cKp and have produced an associated adaptive immune response.

Building on our previous report demonstrating that wild-type, but not *Rag1^-/-^* mice (deficient in both B and T cells), mount a protective recall response against cKp (Twentyman et al., 2020), we hypothesized first that a systemic antibody response was responsible for this protection. However, transfer of convalescent serum from inoculated mice to naïve recipients did not confer protection; in contrast, transfer of hvKp-specific serum mediated strong protection against hvKp challenge. Considering the propensity for dissemination of hvKp, it is likely that transfer of systemic antibody acts to control dissemination from the lung to reduce severity of illness. Conversely, cKp is less prone to dissemination (Rosen et al., 2016) and its pathogenicity may hinge on inflammatory events in the lung, thus rendering a systemic antibody response less effective. The relative importance of antibody-mediated killing by complement or opsonophagocytosis may also contribute to the opposing results observed for these two strains (Loraine et al., 2018; Short et al., 2020). Further experiments will be required to determine the contributions of these factors. Importantly, our serum transfer experimental design does not exclude a requirement for other classes of antibodies in protection against cKp. IgA responses may be more effective than circulating IgG responses against this more localized lung infection (Hall et al., 2021). A phagocytic myeloid population such as alveolar macrophages may also be affected by cKp inoculation and may act in conjunction with antibody responses to clear the challenge infection. These studies do not exclude the possibility that targeted antibody responses raised against specific antigens such as capsule could be protective, but instead indicate that this is not the primary correlate of protection in the wild-type host following cKp lung infection.

We next interrogated the role of T cells and found that mice lacking T cells (*Tcrb*^-/-^*Tcrd*^-/-^) are unable to mount a protective response against challenge. These results indicate that T cells are required for the establishment and/or execution of protection against cKp challenge. Multiple T cell subsets have been demonstrated to mediate protective adaptive immune responses against bacteria in the lung including γδ (Misiak et al., 2017), CD4 (Wilk et al., 2017; Amezcua Vesely et al., 2019), and CD8 (Foxwell et al., 2001) T cells. We identified increased populations of total gamma delta (editor, please convert to greek letters) T cells and total CD4 T cells, as well as increased populations of CD4+CD69+ and CD8+CD69+ T cells in the lung following cKp inoculation. Experiments inhibiting T cell circulation *via* FTY720 showed that additional T cell recruitment to the lung at the time of challenge was not necessary for protection. To determine if the observed lung T cell populations play a role in protection, we performed experiments utilizing mice deficient in only γδ T cells (*Tcrd*^-/-^) or αβ T cells (*Tcrb*^-/-^) including CD4 and CD8 T cells. These studies revealed that each subset alone is sufficient to mediate protection in the absence of the other subset, indicating the protective action is not specific to one lineage but can be performed by either. Of note, these results reflect the use of germline-deficient models lacking T cells throughout the lifetime of the animal. Accordingly, a limitation of these models is the potential for compensation of one cell type in the absence of another, which is not representative of a real-world infection scenario. Novel experimental systems allowing inducible depletion of each subset could confirm if these T cell subsets are truly redundant in this infection, or if one cell type is more effective in the wild-type context.

Finally, we set out to further confirm the T cell requirement by administering anti-CD3 depleting antibody preceding and during inoculation. Mice depleted of T cells at the time of inoculation were rendered susceptible to challenge. These data support the results obtained utilizing genetically deficient mice. The mechanism of T cell protection during cKp reinfection remains a critical unanswered question following these studies. T cells may act on a secondary cell type such as macrophages or B cells that in turn mediate protection during challenge. Alternatively, T cells may act directly by producing IL-17A during challenge, as has been demonstrated in hvKp challenge (Chen et al., 2011). Depletion of T cells at the time of challenge suggested that T cells may not be required at the time of challenge and instead protect *via* action on other cell types; however, these studies were not entirely conclusive due to incomplete depletion of T cells by the antibody-mediated depletion strategy utilized. Surprisingly, mice depleted of T cells at the time of challenge lost less weight during challenge than isotype controls. This potentially reflects inflammation associated with CD3 depletion close to the time of challenge (Ferran et al., 1990).

A limitation in this work is the uncertain degree to which TOP52 represents the spectrum of classical strains, which would affect the generalizability of the pathogenesis and host responses observed. Among classical strains, differences in capsule type and virulence factor expression may lead to significant differences in pathogenesis and the efficacy of immune responses (Gomez-Simmonds and Uhlemann, 2017). Future studies will incorporate additional classical strains to identify features of the host-pathogen interaction that are general versus strain specific.

The results presented here indicate T cells are required during cKp infection in order to establish protective immunity against challenge. These and future results will identify the immune effector functions best suited for clearance of cKp from the lung, informing the design of targeted therapeutic strategies against diverse Kp infections.

## Data availability statement

The raw data supporting the conclusions of this article will be made available by the authors upon request.

## Ethics statement

The animal studies were reviewed and approved by Institutional Animal Care and Use Committee at Washington University School of Medicine.

## Author contributions

JM, CS, and DR conceived and designed the experiments. JM, CMS, RW, JT, and DR performed experiments. JM analysed the data and wrote the original draft of the manuscript. JM and DR edited and reviewed the manuscript. DR acquired funding and supervised the project. All authors contributed to the work and approve of submission.

## Funding

This work was supported by the National Institute of Allergy and Infectious Diseases (K08-AI127714) and by the Children’s Discovery Institute of Washington University. JM was supported through The American Association of Immunologists Careers in Immunology Fellowship Program. JM and CS were supported by The Pediatric Cardiovascular and Pulmonary Research Training Program (5T32HL125241-07).

## Acknowledgments

We thank Drs. David Hunstad, Paeton Wantuch, Teri Hreha, Nicole Gilbert, and Emma Walker for critical feedback on this work and manuscript.

## Conflict of interest

The authors declare that the research was conducted in the absence of any commercial or financial relationships that could be construed as a potential conflict of interest.

## Publisher’s note

All claims expressed in this article are solely those of the authors and do not necessarily represent those of their affiliated organizations, or those of the publisher, the editors and the reviewers. Any product that may be evaluated in this article, or claim that may be made by its manufacturer, is not guaranteed or endorsed by the publisher.
