# Supplementary material for: Classical and γδ T cells are each independently sufficient to establish protection against a classical strain of Klebsiella pneumoniae
Source: Front Cell Infect Microbiol. 2022 Aug 31;12:974175. doi: 10.3389/fcimb.2022.974175 (PMC9471189; doi:10.3389/fcimb.2022.974175)
Supplement: Supplementary file 1 [file DataSheet_1.pdf]

Supplemental Figures

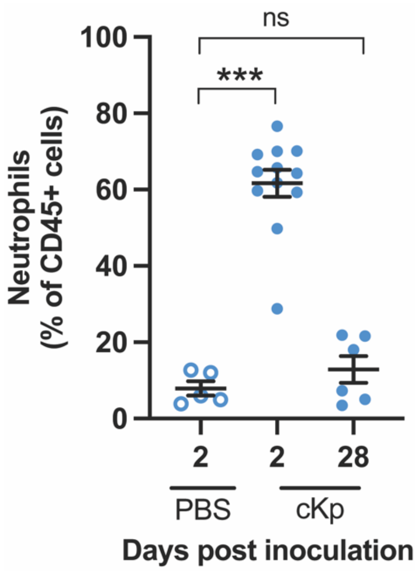

Figure S1. Number of lung neutrophils is increased following cKp infection. Flow cytometric enumeration of CD11b+Ly6G+ neutrophils in the lung 2 days and 28 days following cKp inoculation via oropharyngeal aspiration (10^8 CFU) or 2 days following PBS inoculation (n=5-12 mice per group, 2 independent experiments). Data are expressed as a percentage of total CD45+ cells. Bars indicate mean +/- SEM. *** indicates p<0.001. ns indicates not significant.

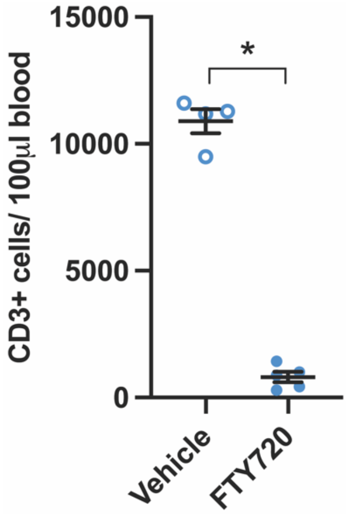

Figure S2. FTY720 treatment decreases T cell numbers in peripheral blood. Flow cytometric enumeration of CD3+ T cells in peripheral blood 24 hours following FTY720 or vehicle treatment (n=4 mice per group, 1 experiment). Data are expressed as T cell number per 100μl blood. Bars indicate mean +/- SEM. * indicates p<0.05.

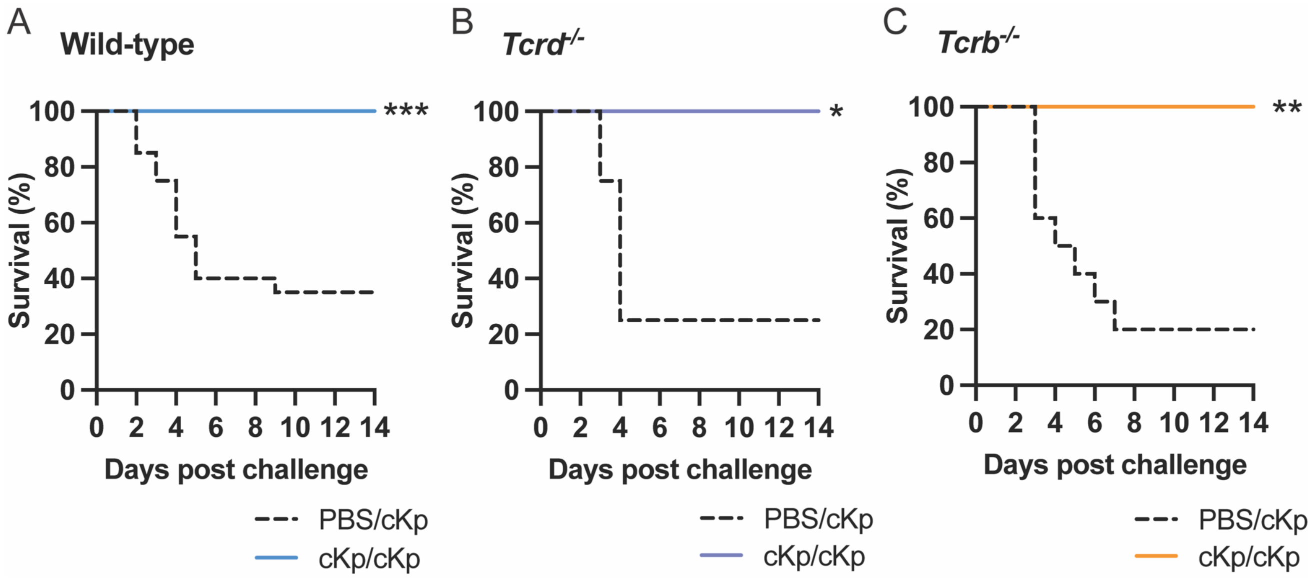

Figure S3. Oropharyngeally inoculated ***Tcrd*** $^{-/-}$ **and** ***Tcrb*** $^{-/-}$ **mice are each protected against high dose cKp challenge.** (A) Survival of WT mice previously-inoculated via oropharyngeal aspiration with PBS or 10^7 CFU cKp over 14 days following challenge with 10^8 CFU cKp (PBS/cKp n=20, cKp/cKp n=20). (B) Survival of *Tcrd*$^{-/-}$ mice previously-inoculated via oropharyngeal aspiration with PBS or 10^7 CFU cKp over 14 days following challenge with 10^8 CFU cKp (PBS/cKp n=4, cKp/cKp n=4). (C) Survival of *Tcrb*$^{-/-}$ mice previously-inoculated via oropharyngeal aspiration with PBS or 10^7 CFU over 14 days following challenge with 10^8 CFU cKp (PBS/cKp n=10, cKp/cKp n=9). Bars indicate mean +/- SEM. *** indicates p<0.001, ** indicates p<0.01, * indicates p<0.05.

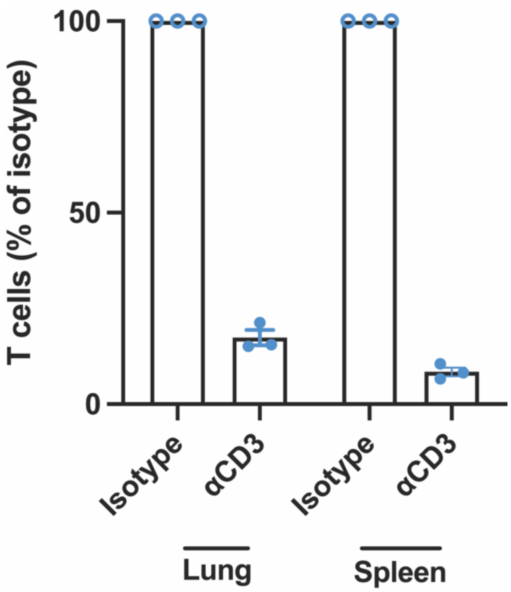

Figure S4. αCD3 antibody treatment reduces T cell number in the lung and spleen. Flow cytometric analysis of lung and spleen T cell numbers on day 0 relative to inoculation following treatment with αCD3 antibody on days -6 and -2. CD4+, CD8+ and TCRγδ+ populations were gated irrespective of CD3 and cell numbers summed. Data are displayed as percent of the average of isotype control animals.

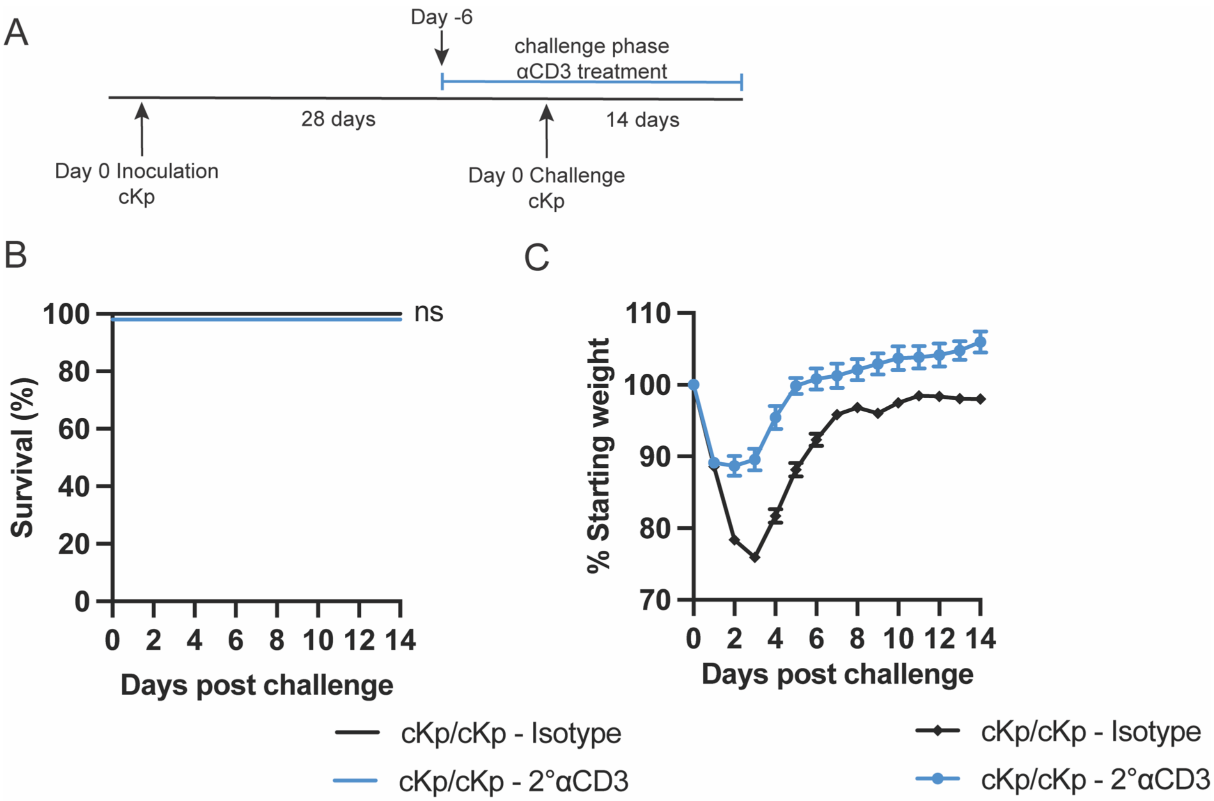

Figure S5. αCD3 antibody treatment prior to challenge does not ablate protection. (A) Scheme of cKp inoculation (10^7 CFU) plus challenge (10^8 CFU) via oropharyngeal aspiration and antibody-mediated T cell depletion in wild-type mice. (B) Survival and (C) weights of anti-CD3 or isotype-treated mice over 14 days following challenge with cKp (n=9-14 mice per group, 2 independent experiments). Weights of anti-CD3-treated mice were significantly higher than weights of isotype-treated mice on days 2-13 post challenge ($p<0.01$). Bars indicate mean +/- SEM. ** indicates $p<0.01$. ns indicates not significant.
